# The Impact of Vitamin D and L-Cysteine Co-Supplementation on Upregulating Glutathione and Vitamin D-Metabolizing Genes and in the Treatment of Circulating 25-Hydroxy Vitamin D Deficiency

**DOI:** 10.3390/nu16132004

**Published:** 2024-06-24

**Authors:** Sushil K. Jain, Jeffrey Justin Margret, Steven A. Abrams, Steven N. Levine, Kamal Bhusal

**Affiliations:** 1 Department of Pediatrics, Louisiana State University Health Sciences Center, Shreveport, LA 71103, USA; jeffrey.justinmargret@lsuhs.edu; 2 Department of Pediatrics and Dell Pediatric Research Institute, Dell Medical School at the University of Texas at Austin, Austin, TX 78723, USA; sabrams@austin.utexas.edu; 3 Department of Medicine, Louisiana State University Health Sciences Center, Shreveport, LA 71103, USA; steven.levine@lsuhs.edu (S.N.L.); kamal.bhusal@lsuhs.edu (K.B.)

**Keywords:** 25(OH)VD deficiency, African American, androgenic index, GSH, H_2_S, inflammation, L-cysteine, NO, SHBG, vitamin D

## Abstract

Vitamin D receptors are expressed in many organs and tissues, which suggests that vitamin D (VD) affects physiological functions beyond its role in maintaining bone health. Deficiency or inadequacy of 25(OH)VD is widespread globally. Population studies demonstrate that a positive association exists between a high incidence of VD deficiency and a high incidence of chronic diseases, including dementia, diabetes, and heart disease. However, many subjects have difficulty achieving the required circulating levels of 25(OH)VD even after high-dose VD supplementation, and randomized controlled clinical trials have reported limited therapeutic success post-VD supplementation. Thus, there is a discordance between the benefits of VD supplementation and the prevention of chronic diseases in those with VD deficiency. Why this dissociation exists is currently under debate and is of significant public interest. This review discusses the downregulation of VD-metabolizing genes needed to convert consumed VD into 25(OH)VD to enable its metabolic action exhibited by subjects with metabolic syndrome, obesity, and other chronic diseases. Research findings indicate a positive correlation between the levels of 25(OH)VD and glutathione (GSH) in both healthy and diabetic individuals. Cell culture and animal experiments reveal a novel mechanism through which the status of GSH can positively impact the expression of VD metabolism genes. This review highlights that for better success, VD deficiency needs to be corrected at multiple levels: (i) VD supplements and/or VD-rich foods need to be consumed to provide adequate VD, and (ii) the body needs to be able to upregulate VD-metabolizing genes to convert VD into 25(OH)VD and then to 1,25(OH)2VD to enhance its metabolic action. This review outlines the association between 25(OH)VD deficiency/inadequacy and decreased GSH levels, highlighting the positive impact of combined VD+LC supplementation on upregulating GSH, VD-metabolizing genes, and VDR. These effects have the potential to enhance 25(OH)VD levels and its therapeutic efficacy.

## 1. Introduction

Vitamin D (VD) is an essential nutrient that helps maintain healthy bones [1]. Many organs and tissues express vitamin D receptors, suggesting that vitamin D affects physiological functions beyond its role in maintaining bone health [1,2,3] and is crucial for the regulation of numerous important genes [4]. Vitamin D deficiency affects nearly one billion individuals globally. In the US, nearly 45% of the population is vitamin D deficient. In African Americans (AAs), 70% are vitamin D deficient compared with only 25% of the white population [5]. Epidemiological research has shown a link between vitamin D deficiency and a greater incidence of chronic diseases, including heart disease and diabetes [6,7,8]. The escalating incidence of 25(OH)vitamin D (25(OH)VD) deficiency/inadequacy has led to increased awareness and use of vitamin D supplements by the general population and more prescriptions for vitamin D by physicians [9]. However, randomized controlled clinical trials have reported limited therapeutic success after supplementation with vitamin D [10,11,12,13]. Thus, there is a dissociation between the benefits of vitamin D supplementation and the prevention of chronic diseases in those with vitamin D deficiency. Why this disconnect exists is currently under debate. Vitamin D deficiency is widespread despite the availability of vitamin D obtained through sunlight, food, and supplements [1]. This review discusses how those with metabolic syndrome, obesity, and other chronic diseases exhibit insufficient glutathione (GSH) levels and downregulation of the vitamin D-metabolizing genes required to convert consumed vitamin D into 25(OH)VD and enable its metabolic action [10,11]. It has become clear that vitamin D deficiency needs to be corrected at multiple levels: (i) vitamin D supplements and/or vitamin D-rich foods need to be consumed to provide adequate vitamin D, (ii) the body needs to be able to upregulate vitamin D-metabolizing genes to convert vitamin D into 25(OH)VD and then to 1,25(OH)_2_VD, and (iii) the levels of inflammatory biomarkers need to be reduced.

## 2. VD Metabolism Genes and Blood 25(OH)VD Status in Humans

**Figure 1:**
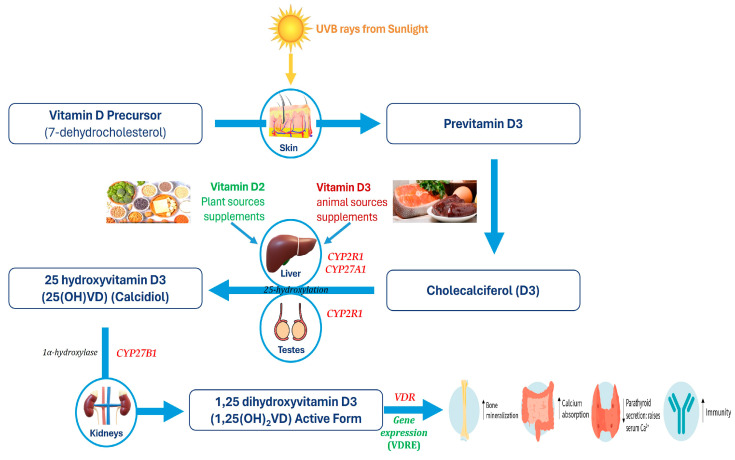
Vitamin D metabolic pathway.

The primary sources of cholecalciferol or vitamin D in humans are dermal synthesis and diet. The biosynthesis of vitamin D in the human body is stimulated by skin exposure to ultraviolet B rays from sunlight. Cholecalciferol (VD) is converted to 25(OH)VD by VD-25-hydroxylase (*CYP2R1*, *CYP27A1*) in the liver [14,15]. Then, 25(OH)VD is bound to VD-binding protein (*VDBP*) and transported into the circulation. While the liver is the main site of VDBP synthesis and secretion [16,17], the conversion of 25(OH)VD to its active metabolite, 1,25(OH)_2_VD (calcitriol), is carried out by *CYP27B1* in both renal and non-renal tissues [18] (Figure 1). *CYP24A1* is responsible for the catabolic inactivation of 25(OH)VD and 1,25(OH)_2_D, which helps regulate 1,25(OH)_2_D signaling [19]. Study of the mutations in *VDBP/CYP2R1* genes in humans and *Vdbp/Cyp2r1* knockdown mice models showed low levels of 25(OH)VD [14,20]. The bioavailability of 25(OH)VD in response to ingesting vitamin D supplementation significantly varies among individuals and is influenced by the status of the vitamin D-metabolizing genes [16,21,22,23]. The levels of 1,25(OH)_2_VD are regulated by the circulating PTH concentrations [24], while the biological actions of 1,25(OH)_2_VD depend on the status of the VDR in the target tissues [25]. Here, 1,25(OH)_2_VD binds to VDR and translocates to the nucleus, regulating target gene transcription. Thus, biosynthesis of 25(OH)VD and 1,25(OH)_2_VD and downstream actions of the *VDR* (VDR/PGC-1α/GLUT4) are under the control of the VD regulatory genes (Figure 1). The levels of the stable metabolite 25(OH)VD can be measured to diagnose 25(OH)VD deficiencies and monitor the consumption of vitamin D [26]. Upregulation of vitamin D regulatory genes can beneficially increase 25(OH)VD levels and the metabolic actions of 1,25(OH)_2_VD [27,28].

The risk factors for 25(OH)VD deficiencies include race, elevated BMI, winter season (fewer daylight hours), living in regions with higher latitudes, and consuming diets lacking in vitamin D [29,30]. According to the National Center for Health Statistics, people with darker skin are at risk for 25(OH)VD inadequacy (41%) and deficiency (32%) [31]. The incidence of 25(OH)VD deficiency/inadequacy is increasing due to the rise in metabolic syndromes such as insulin resistance (IR), obesity, and diabetes. AAs tend to experience much higher levels of 25(OH)VD deficiency and IR [32,33].

## 3. Bioavailable 25(OH)VD Is Linked with Better Health Outcomes

Approximately 85–90% of 25(OH)VD is bound tightly to VDBP [13,34]. Moreover, 25(OH)VD and free 25(OH)VD, which is loosely bound to albumin, are the two distinct pools of total 25(OH)VD. The free hormone hypothesis proposes that hormones not bound to carrier proteins with high affinity can freely diffuse across cell membranes to perform their biological activities [13,34]. Bioavailable 25(OH)VD is the sum of the free and albumin-bound vitamin D. Various clinical investigations have demonstrated that bioavailable 25(OH)VD serves as a better biomarker for assessing vitamin D levels and a predictor of its health outcomes compared to total 25(OH)VD [35,36].

## 4. Impaired Vitamin D-Metabolizing Genes in Obesity/Population

A significant shift in the global work culture, limited outdoor activities and/or exercise, lack of healthy food consumption, and in some populations, the presence of darker skin [29] lead to limited consumption of dietary cholecalciferol and limited endogenous production of cholecalciferol from 7-dehydrocholesterol. Those with obesity and metabolic syndrome have a higher prevalence of vitamin D deficiency and inflammation. The efficacy of consumed vitamin D depends on the genes that are required to convert vitamin D to 25(OH)VD metabolite and to 1,25(OH)_2_VD metabolite. The biological action of consuming vitamin D depends upon active vitamin D at various cellular levels. Table 1 summarizes multiple studies carried out in human peripheral blood mononuclear cells. These studies from different laboratories report a downregulation or depression in the levels of vitamin D-metabolizing genes in subjects with metabolic syndrome and obesity. Specific mechanisms proposed for this depressed level of vitamin D-metabolizing genes are hypermethylation of specific sites of genes and elevated BMI [37,38]. Table 2 summarizes multiple studies using obese animal models investigating vitamin D-metabolizing gene expression in the liver and other tissues. These studies report an association between obesity-elevated inflammatory biomarkers and depressed levels of vitamin D-metabolizing genes. These genes are required to convert consumed vitamin D into 25(OH)VD to enable its metabolic action [10,11].

## 5. Impaired Glutathione and Obesity

Obesity, a prevalent health challenge in the modern era, is a chronic condition that impacts the physical, financial, and psychological well-being of individuals, regardless of their cultural, financial, or ethnic context. Obesity arises from multiple factors and is characterized by an excessive buildup of body fat [52]. Excessive body fat diminishes quality of life, raises healthcare expenses, and increases the mortality risk. Obesity is linked to various health issues, such as diabetes, heart problems, cancer, asthma, sleep apnea, liver and kidney dysfunction, as well as infertility [53].

The pathogenesis of obesity and its associated risk factors have been studied extensively through epidemiological, clinical, and animal research studies, all of which have consistently highlighted the significant role of oxidative stress in this process [53]. Oxidative stress has the potential to induce obesity through the accumulation of white adipose tissue (WAT) and changes in food consumption. Research involving cell cultures and animal models has shown that oxidative stress can lead to increased preadipocyte proliferation, enhanced adipocyte differentiation, and enlargement of mature adipocytes [52,54]. Reactive oxygen species (ROS) regulate body weight by affecting hypothalamic neurons, which control satiety and hunger [53]. Obesity has the potential to induce systemic oxidative stress through multiple biochemical pathways. These pathways involve the production of superoxide by NADPH oxidases, oxidative phosphorylation, glyceraldehyde auto-oxidation, activation of protein kinase C, and the participation of polyol and hexosamine pathways. Additionally, other factors, such as hyperleptinemia, tissue dysfunction, compromised antioxidant defense, chronic inflammation, and postprandial ROS generation, also contribute to oxidative stress in individuals with obesity [53].

Oxidative stress has a negative impact on both bone tissue quality and bone catabolism [55]. Glutathione (GSH) depletion increases oxidative stress and extensive carbonylation of proteins [56,57,58]. Endogenous enzymes and proteins can be covalently modified through oxidative modification or carbonylation, leading to protein dysfunction, impaired cell function, and contributing to the etiology of various human diseases [53,59,60,61,62]. Supplementation with GSH or L-cysteine (LC, a GSH precursor) has been effective in enhancing GSH levels in blood and tissues, reducing inflammation and insulin resistance in both humans and animals [63,64,65]. Inadequate levels of GSH can elevate oxidative stress, resulting in increased inflammatory markers like TNF-α, disruption of enzyme and protein function, and insulin resistance [10,11,44,66,67]. The association between oxidative stress and obesity becomes stronger as the BMI increases [68].

GSH plays a crucial role as an antioxidant and acts as a co-factor for numerous enzymes. The levels of GSH in the bloodstream serve as an indicator of the body’s ability to combat oxidative stress and maintain its defense mechanisms against it [44,47,69,70]. Exhausted or impaired antioxidant pathways in obese individuals and mice fed a high-fat diet are indicated by decreased levels of GSH in the blood and increased oxidative stress. The lower levels of GSH can be attributed to various factors, such as a lack of LC in the diet, increased production of ROS and oxidative stress caused by the consumption of an energy-rich diet, and/or increased utilization of GSH compared to its biosynthesis. It has been observed that the blood levels of GSH are lower in obese, diabetic, and AA subjects [32,33,44,70,71,72,73,74,75]. The formation of GSH occurs through the enzymatic action of glutamate-cysteine ligase (GCL) and glutathione synthetase [58,76]. NRF2 transcription factor is also implicated in the regulation of the GSH biosynthesis genes (GCLC, GCLM). The MDA assay measures the reactive aldehydes formed during lipid peroxidation, such as malondialdehyde and 4-hydroxynonenal. The protein carbonyl assay measures protein carbonyl derivatives formed during the oxidation of specific amino acids in proteins or introduced into proteins by a secondary reaction of nucleophilic side chains to amino acids, such as LC residues, with aldehyde products of lipid peroxidation [57]. Protein-bound carbonyls represent an irreversible form of protein modification and are relatively more stable in contrast to lipid peroxidation products [77]. Oxidative modification or carbonylation of proteins leads to the covalent alteration of endogenous enzymes and proteins, which can cause a loss of protein function, disrupt metabolism, and impair cellular function [56,78].

### Link between GSH and 25(OH)VD

Studies of obesity and diabetes in humans, as well as in experimental animals that consumed a high-fat diet (HFD), have shown elevated oxidative stress and impaired GSH status [53,54,59,70]. Blood analyses of similarly aged healthy children, in a group comprising lean, overweight, and obese subjects, showed significantly lower levels of 25(OH)VD and GSH in obese (BMI > 30) compared with lean (≤25 BMI) or overweight (≤30 and >25 BMI) subjects; in addition, a significant positive relationship was seen between the blood level of 25(OH)VD and that of GSH. A study carried out in healthy adolescents, with a much wider age range among the subjects, ruled out the role of confounding variables, such as diabetes, medications, and age, in the association between GSH and 25(OH)VD. Previous studies have reported a positive association between the concentrations of GSH in the blood and 25(OH)VD in adults, children, and diabetic subjects [71,72,74]. The blood levels of 25(OH)VD are independently associated with GSH and redox status in adults [74]. Studies have demonstrated a connection between the levels of serum vitamin D and the overall antioxidant capacity in both diabetic adults and obese adolescents [79,80]. The intake of dietary antioxidants has been found to have a positive impact by elevating the levels of serum 25(OH)VD [81].

Silencing of glutamate-cysteine ligase (GCLC), a rate-limiting enzyme in GSH biosynthesis, caused an increase in oxidative stress/protein oxidation and downregulation of the the *CYP2R1*, *CYP27A1*, *CYP27B1*, *VDBP*, and *VDR* genes in cultured hepatocytes. *GCLC* knockdown (GCLC KD) resulted in simultaneous downregulation of GSH and the mRNA levels of *CYP27A1*, *CYP27B1*, *VDBP*, and *VDR* in hepatocytes. GSH deficiency impairs the expression of vitamin D regulatory genes, but supplementation with VD and LC can improve the levels of GSH and vitamin D regulatory genes in liver cells [82]. GSH positively enhances the expression of *CYP27B1*, leading to the conversion of 25(OH)VD to 1,25(OH)_2_VD [44,47]. While the kidney is traditionally known as the primary site for 1,25(OH)_2_VD production, recent research reveals the presence of *CYP27B1* in non-renal cells and tissues, suggesting localized formation of 1,25(OH)_2_VD and its tissue-specific paracrine role across various tissues [7]. Most cells express *VDR*, and its expression can be modulated by the GSH status [25,69]. The mechanism of the biological actions of 1,25(OH)2VD involves heterodimeric complex formation between 1,25(OH)_2_VD and VDR/RXRα [83,84,85]. The VDR content of target tissues directly influences the biological actions of 1,25(OH)_2_VD. Once bound to VDR, 1,25(OH)_2_VD translocates to the nucleus and controls the transcription of target genes. Physiological factors such as Ca^2+^, 25(OH)VD, 1,25(OH)_2_VD, and *VDBP* regulate the expression of *VDR* [24].

Various studies in humans and animals have successfully used LC, *N*-acetyl-L-cysteine (NAC), and/or LC-rich whey protein supplementation to improve the status of GSH and lower the levels of inflammation and insulin resistance in blood and tissues [63,64,86,87,88,89,90]. Evidence in the literature indicates a strong correlation between increased intake of dairy products and leafy greens and biomarkers of bone health and levels of 25(OH)VD in the bloodstream [91]. Milk and leafy vegetables contain high levels of vitamin D, glutathione, and methionine/LC, which could potentially enhance vitamin D absorption and overall well-being through their consumption. Improvement of the GSH status using LC supplementation resulted in the upregulation of genes in both hepatocytes and myotubes [69].

Similar to the reduced GSH levels seen in obese adolescents, the GSH concentrations were also reduced in the blood, liver, and muscle tissues of mice that consumed an HFD compared with those of mice that consumed a normal diet. Interestingly, the tissues of the HFD-fed mice showed a significant decrease in the mRNA levels of the GSH-biosynthesis genes (*NRF2*, *GCLC*, and *GCLM*), VD regulatory genes (*CYP2R1*, *CYP27A1*, *CYP27B1*, *VDBP*, and *VDR*) in the liver, and of GSH-metabolism genes and GLUT4 gene transcription factors (VDR/PGC-1α) in the skeletal muscle compared to those of mice that consumed a normal diet [44,47]. GLUT4 is considered a master regulator of glucose metabolism. LC enhances the expression of vitamin D regulatory genes and contributes to the activation of GLUT4. The protein oxidation and lipid peroxidation levels were notably decreased in the liver and muscle of mice co-supplemented with VD+LC compared to those supplemented with only vitamin D. An increased GSH status may help mitigate oxidative stress induced by HFD intake. Combined supplementation with VD+LC caused a significant upregulation of GSH-synthesizing enzymes, GSH, and vitamin D regulatory genes in both liver and muscle. There was significant upregulation of PGC-1α, NRF2, and GLUT4 in muscle from mice supplemented with VD+LC. Co-supplementation of VD+LC in mice resulted in elevated NRF2, increasing the antioxidant enzymes and improving the cellular glutathione levels. Consequently, this supplementation effectively reduced the oxidative stress levels in tissues, as compared to mice supplemented with vitamin D alone [44,69,70].

**Figure 2:**
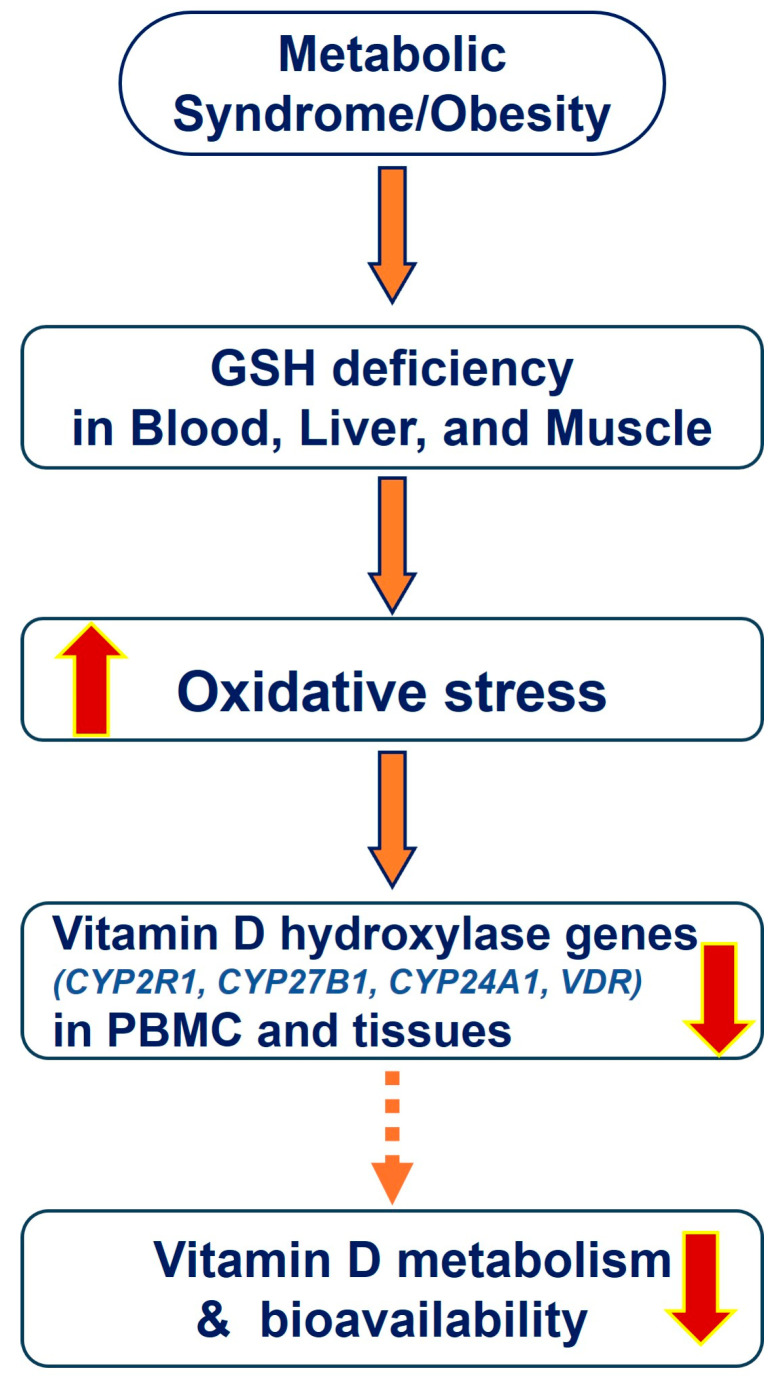
The proposed mechanism by which GSH deficiency downregulates VD hydroxylases and metabolism genes and the bioavailability of VD.

The efficacy of VD in raising blood 25(OH)VD and GSH and reducing the inflammation levels in vitamin D-deficient mice was significantly greater when vitamin D was supplemented in combination with LC, in comparison with supplementation with vitamin D alone. The potential mechanism responsible for the increasing blood levels of 25(OH)VD could be that the improvement in the GSH status reduces oxidative stress and upregulates vitamin D-metabolizing genes, thereby increasing blood levels of 25(OH)VD (Figure 2). Furthermore, the translocation of the VDR/1,25(OH)2VD complex is induced by the upregulation of VDR expression in target tissues, making it available for metabolic action, such as GLUT4 upregulation. Thus, LC not only improves the GSH status, which enables the upregulation of vitamin D regulatory genes, but also reduces the TNF-α levels and adds to VDR/PGC-1α/GLUT4 activation. Therefore, combined supplementation with LC along with vitamin D can stimulate the levels of GSH, thus helping reduce the 25(OH)VD deficiency/inadequacy and inflammation associated with obesity and type 2 diabetes [44,69,73].

The association between the GSH and 25(OH)VD statuses is unlikely to be non-specific [1]. In human studies, this relationship was observed in non-diabetic persons who were healthy and not taking any medication as well as in diabetic patients who were not healthy and were on medications [2]. In cell culture studies, the knockdown of specific enzymes that synthesize GSH did cause both a decrease in GSH and the VD metabolism genes statuses, and L-cysteine supplementation simultaneously caused an improvement in both GSH and VD metabolism genes [3]. In animal studies, the high-fat diet-induced onset of obesity simultaneously caused a decrease in both GSH and the 25(OH)VD blood levels. In addition, previous studies have reported that compared with the summer season, the blood levels of GSH and 25(OH)VD are lower in the winter season in the same subject [80,92,93].

These studies demonstrate a novel pathway through which the status of GSH can enhance the levels of 25(OH)VD and that the use of combined VD+LC supplementation significantly lowers inflammation and increases the levels of GSH, vitamin D regulatory genes, and VDR, all of which are required to raise the blood levels of 25(OH)VD and reduce the inflammation levels. The combined use of VD and LC provides a novel approach to stimulate vitamin D regulatory genes and protects against 25(OH)VD deficiency [70,71].

## 6. LC, GSH Biosynthesis, Oxidative Stress, and Inflammation

L-cysteine is semi-essential and can be synthesized by the body under normal physiological conditions if a sufficient quantity of methionine is available. The gastrointestinal (GI) tract breaks down dietary LC into cystine. Cystine then safely passes through the GI tract and blood plasma and is quickly converted into two LC molecules upon cell entry. The enzymes glutamate-cysteine ligase and glutathione synthetase play a crucial role in the formation of GSH from LC, glycine, and glutamate [58]. LC is considered a rate-limiting precursor of glutathione biosynthesis and a physiological antioxidant and anti-inflammatory molecule. The reduced form of glutathione is crucial to protecting the body from oxidative stress-induced damage. It can counteract reactive particles that can harm cells and tissues. Therefore, supplementing the diet with LC can restore glutathione synthesis in compromised cases, leading to an improved redox balance and reduced oxidative stress [94]. Low levels of GSH or LC may lead to elevated levels of ROS and oxidative stress, impaired reduction of oxidized GSSG to GSH, and/or heightened consumption of GSH compared to its production [63,86,89,95,96].

A reduced GSH status can further compromise the defense against oxidative stress and increase the oxidative modification of proteins or enzymes, causing major changes in the secondary structure that result in the impaired metabolic stability and function of modified proteins or enzymes [78,97]. Supplementation with a combination of glycine and *N*-acetylcysteine (a cysteine precursor) has been shown to enhance and rectify deficiencies in cellular glycine, cysteine, and GSH. Additionally, it has been found to reduce oxidative stress, improve mitochondrial function, alleviate inflammation, decrease IR, and target various hallmarks of aging [87].

LC transporter (*SLC7A10*) mRNA in adipose tissue shows a strong inverse correlation with IR, adipocyte size, and metabolic syndrome components, along with a strong heritability and an association with type 2 diabetes risk alleles. Overexpression of *SLC7A10* in mature white adipocytes was observed to reduce ROS generation and enhanced suppression of *SLC7A10* had the opposite impact, suggesting that *SLC7A10* supports a beneficial increase in mitochondrial activity within white adipocytes [98]. Nuclear factor erythroid-2-related factor (NRF2) is implicated in the biosynthesis of GSH [99,100,101] and protection from oxidative stress and tissue damage [101]. The levels of peroxisome proliferator-activated receptor gamma coactivator-1 alpha (PGC-1α) and NRF2 are reduced in human tissues in obesity and diabetes [102]. PGC-1α upregulates the expression of GLUT4 in skeletal muscle [103,104,105], inhibits pro-inflammatory cytokine production [106], and is a co-activator of the retinoic X receptor (RXRα) [83,85,107]. The genomic mechanism of 1,25(OH)_2_VD action involves the direct binding of the 1,25(OH)_2_VD activated VDR/RXRα heterodimeric complex to specific DNA sequences [83,84,85]. PGC-1α functions as a co-factor for many transcription factors, including NRF2 [102]. Animal studies suggest that LC supplementation has the potential to upregulate PGC-1α/NRF2 and reduce IR [108].

These animal and cell culture studies suggest that the improvement in GSH status that results from co-supplementation with VD and LC had significant positive results compared with vitamin D alone in ZDF rats and in a mouse model of 25(OH)VD deficiency. The liver exhibited an increase in VD regulatory genes (VDBP/VD-25-hydroxylase/VDR) and the muscle showed an upregulation of glucose metabolism genes (VDR/PGC-1α/GLUT-4). Additionally, there was an increase in the 25(OH)VD levels in the blood and a decrease in IR [69,109]. GSH deficiency in cell culture studies induced oxidative stress, leading to the downregulation of VDBP/VD-25-hydroxylase/VDR, and upregulation of *CYP24A1* in hepatocytes. Additionally, the downregulation of PGC-1α/VDR/GLUT-4 was observed in myotubes [44]. GSH deficiency epigenetically altered the vitamin D biosynthesis pathway genes in the livers of diabetic mice [47]. The data from these studies provide evidence of a novel mechanism that connects 25(OH)VD deficiency/inadequacy and lower GSH levels. These findings emphasize that the commonly consumed vitamin D supplements may not be effective unless the GSH levels are increased to enhance the function of the vitamin D-metabolizing genes. Therefore, a more effective approach to improve bioavailability and increase blood levels of 25(OH)VD would be to consume both LC and VD nutrients together rather than relying solely on high-dose vitamin D supplementation. This approach is both innovative and superior in achieving desired outcomes in response to vitamin D consumption.

## 7. Testosterone and Vitamin D Metabolism

Testosterone in men upregulates the vitamin D-metabolizing genes, which increases the total vitamin D. Cell culture studies have shown that testosterone treatment of monocytes upregulates vitamin D-metabolizing genes, which can contribute to elevated vitamin D levels in men compared to women [110]. Previous studies report that the testes also have high levels of *CYP2R1*, which can promote vitamin D hydroxylation in men [110,111,112,113]. This may suggest that adequate levels of vitamin D are required to optimize the effect of testosterone in vitamin D-deficient men. The free/total testosterone ratio (androgenic index) has been used to assess the influence of testosterone on metabolic pathways [110,114,115,116]. Traditionally, sex hormone-binding globulin (SHBG) has been considered a binding protein that transports testosterone and estradiol to target tissues and regulates the free concentration of testosterone and estradiol. However, SHBG also influences biological actions independent of total or free testosterone [117,118]. Serum SHBG can directly mediate steroid hormone signal transduction at the plasma membrane. SHBG prevents sex steroid deficiency by increasing its absorption, half-life, and steroid biosynthesis [119]. Deficient SHBG may contribute to the pathogenesis of inflammation by modulating the biological effects of sex hormones (testosterone and estrogen) on peripheral tissues (liver, muscle, and fat) [120,121]. Previous studies in transgenic mice that overexpress human SHBG transgenes have shown that they circumvent metabolic syndrome, inflammation, and type 2 diabetes [121,122]. Studies have concluded that SHBG suppresses inflammation and acts on macrophages, muscles, and adipocytes [123].

Co-supplementation of alpha-lipoic acid with NAC has prevented intensive swimming-induced testicular spermatogenic and steroidogenic disorders by decreasing ROS generation [124]. Modification of the Nrf2/HO-1 signaling pathway is one of the mechanisms through which NAC, a powerful antioxidant, exerts significant protective effects against busulfan-induced male reproductive impairment [125]. NAC protects against chromium-induced oxidative damage in mice testes [126] and the testes of rats treated with sodium fluoride by reducing lipid peroxidative 8-hydroxy-2-deoxyguanosine formation [127].

NAC attenuates the blood–testis barrier damage caused by the SR X-ray [128] and may be used as a preventive measure against iron overload-induced testicular damage [129]. The antioxidant effect of NAC reduces the damage caused by various chemicals and radiation to testicular cells [130,131,132,133]. NAC is a well-tolerated mucolytic drug that decreases the viscosity of mucous secretions and enhances glutathione S-transferase activity. NAC possesses strong antioxidant properties and shows a potential therapeutic intervention for conditions marked by the production of free oxygen radicals. Its effectiveness as an antioxidant is attributed to its ability to serve as a precursor to glutathione, a key endogenous antioxidant in the body [12]. Oral supplementation with NAC improves sperm parameters and reduces oxidative stress in infertile men [134].

## 8. L-Cysteine, Nitric Oxide, Hydrogen Sulfide, and Vitamin D Metabolism

Nitric oxide (NO) is a gaseous signaling molecule crucial for maintaining vascular homeostasis. The synthesis of NO occurs when L-arginine is converted by nitric oxide synthases (NOS) in the presence of oxygen [135]. The plasma nitrite levels, a well-known indicator of NO production, exhibit a higher concentration in healthy individuals during the summer compared to the winter. This disparity could be attributed to increased exposure to UV-A radiation, which triggers the release of NO metabolites from the skin. Furthermore, it is plausible that the fluctuation in nitric oxide availability throughout the seasons contributes to elevated blood pressure during winter [135]. Reduced NO synthesis is linked to both aging and VD deficiency [136]. Hydrogen sulfide (H_2_S), which is produced in vivo from LC catalyzed by the enzyme CSE [137], plays a crucial role in regulating numerous cellular functions and biochemical processes. Several reviews discuss the potential benefits of NO and H_2_S availability in biological systems and the association of decreased levels with the development of cardiovascular diseases and an increased risk of pathogenic events [138,139,140].

Vitamin D can regulate the production of NO and/or the expression of inducible NOS (iNOS) in various types of cells, such as endothelial cells, osteoblasts, microglial cells, macrophages, and astrocytes [140]. Vitamin D acts as a transcriptional regulator for eNOS, leading to enhanced production of NO, which is known as the most powerful vasodilator in the vasculature [141]. The bioavailability of NO is reduced in VDR knockout mice, leading to an increase in arterial stiffness [142]. Endothelial dysfunction and the compromised production of endothelial-dependent NO are the key factors linking vitamin D deficiency to cardiovascular disease [143]. The potential benefits of vitamin D include enhancing endothelial function and promoting the production of endothelial nitric oxide synthase (eNOS), as well as reducing inflammation-induced endothelial dysfunction [144]. NO deficiency contributes to the pathogenesis of various neurological diseases related to reproduction, inflammation, vasodilation, and cardiac function [145].

Vitamin D plays a role in regulating the synthesis of NO by influencing the activity of endothelial NO synthase (eNOS) in the endothelial cells. In pathological conditions, excessive production of ROS leads to oxidative stress, which promotes the degradation of NO and inhibits its synthesis, resulting in reduced NO bioavailability. Vitamin D counteracts the activity of nicotinamide adenine dinucleotide phosphate (NADPH) oxidase, which is responsible for ROS production, and enhances the antioxidant capacity by increasing the activity of antioxidative enzymes such as superoxide dismutase [146]. Supplementation with L-arginine and beetroot extracts rich in nitrates elevated the vitamin D levels in individuals aged 60 and above at risk of sarcopenia who also participated in a physical activity regimen [147].

LC is unique in that it can upregulate the levels of both NO and H_2_S. The potent scavenging action of H_2_S on peroxynitrite implies a chemical interplay between H_2_S and NO/reactive nitrogen species. The ability of H_2_S to effectively remove peroxynitrite suggests a potential chemical interaction between H_2_S and NO/reactive nitrogen species [148]. In the vascular system, H_2_S regulates the availability of NO [149]. The production of NO enhances the accessibility of nutrients and hormones by causing blood vessels to dilate, thereby increasing their bioavailability [150]. Supplementation using NO precursors such as L-arginine and beetroot extracts, as well as LC, resulted in a significant increase in circulating 25(OH)VD levels and a decrease in oxidative stress and inflammation [147]. Our previous study showed that H_2_S and NO_2_ treatment upregulated the relative expression of CYP2R1 and CYP27B1 genes in THP-1 monocytes [151]. Cell culture and animal studies findings report increased levels of 25(OH)VD in LC-supplemented animals and humans supplemented with NO precursors such as L-arginine, suggesting that elevated levels of H_2_S and NO can increase the bioavailability of vitamin D and blood levels of 25(OH)VD.

## 9. Justification for Combined Use of VD and LC

**Figure 3:**
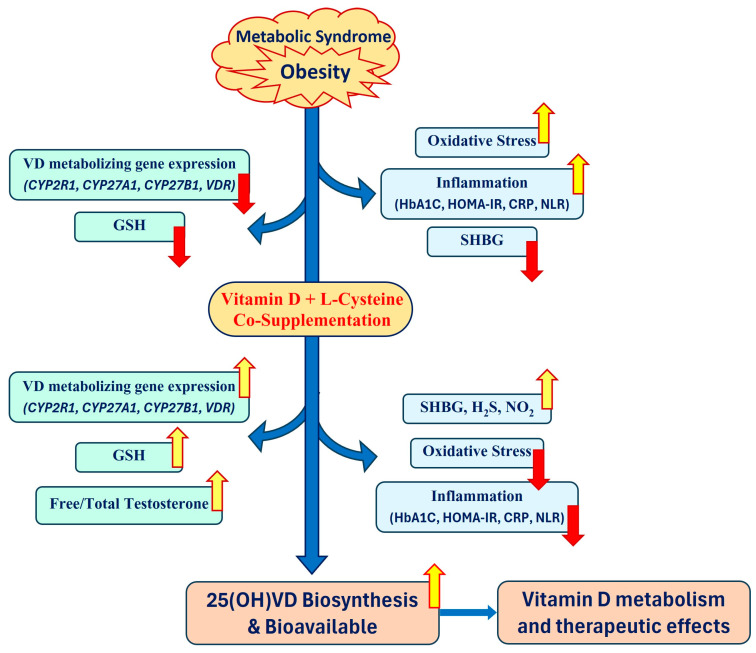
A novel mechanism highlighting combined VD+LC supplementation, which is hypothesized to reduce IR and inflammation biomarkers and increase blood levels of 25(OH)VD, thereby increasing VD metabolism and its therapeutic effects. Yellow arrow, upregulation; red arrow, downregulation.

Recent studies indicate a positive correlation between the blood levels of GSH and those of 25(OH)VD in normal adults, AA type 2 diabetics, and children [70,71,74]. However, no previous study has investigated the effect of improving the GSH status by combined supplementation with VD and LC on the levels of vitamin D regulatory proteins and 25(OH)VD (mechanistic signatures) and a simultaneous decrease in IR (biological signatures). Increasing GSH with LC supplementation has demonstrated a positive effect on insulin sensitivity in clinical trials [63,152]. The central hypothesis is that LC upregulates the synthesis of GSH and the status of vitamin D regulatory genes and thereby increases the 25(OH)VD and 1,25(OH)_2_VD levels and its metabolic action, such as GLUT4 upregulation. In addition, LC induces PGC-1α/GLUT4 upregulation independent of vitamin D. Thus, LC not only upregulates vitamin D regulatory genes and the 25(OH)VD status but also adds to PGC-1α/GLUT4 activation, substantially decreasing and possibly preventing IR. Upregulation of the vitamin D-metabolizing genes using combined VD+LC supplementation, thereby increasing the blood levels of 25(OH)VD and reducing IR and inflammation biomarkers, is a highly innovative approach (Figure 3). The mechanism is potentially responsible for the increased blood levels of 25(OH)VD and the reduction in IR in combined VD+LC-supplemented animals and may result from an improved GSH status. It thereby reduces oxidative stress and upregulation of *VDBP/CYP2R1/CYP27A1/VDR*, which is required for the efficient transport and hydroxylation of cholecalciferol, and activation of the VDR/PGC-1α/GLUT-4 pathway responsible for the metabolic actions of 1,25(OH)_2_VD [44]. It is essential for the VD-hydroxylase/metabolism genes required for the conversion of vitamin D to 25(OH)VD for effective use by the body [1,44,71]. Animal studies have shown that consumption of VD and LC is more effective in raising blood levels of 25(OH)VD (treating vitamin D deficiency) and lowering IR and inflammation compared to intake of vitamin D alone [82,153]. These findings focus attention on the fact that vitamin D supplements are unlikely to be successful unless the status of the vitamin D and vitamin D-metabolizing genes is also corrected by improving the GSH status.

However, higher doses of LC could result in a greater number of side effects being experienced. These include, but are not limited to, sleepiness, intestinal gas, indigestion, dysphoria, local erythema, swelling, lightheadedness, nausea, rashes, and coughing [154]. Similarly, an overdose of VD may also cause adverse health effects [155]. The rise in awareness regarding vitamin D deficiency and its impact on health has led to a significant increase in the use of vitamin D supplements. The excessive use of vitamin D supplements without proper medical consultation can lead to vitamin D toxicity [156]. The suggested maximum safe intake of cholecalciferol is 4000 IU daily [155]. Vitamin D overdose may cause hypercalcemia, vomiting, polydipsia, dehydration, constipation, pain, loss of appetite, and cardiovascular and renal complications [156,157]. Therefore, it may be advisable to consider a daily intake of 1000 mg of LC combined with 2000 IU of VD as a potentially safer approach to elevate vitamin D levels in the bloodstream.

## 10. Conclusions

Deficiencies in 25(OH)VD are widespread globally. Substantial data in the literature support the role of vitamin D deficiency in the development of chronic health problems. Preclinical studies suggest that the simultaneous intake of LC and VD nutrients, rather than solely using high doses of vitamin D, represents an innovative and better approach to enhancing the bioavailability of cholecalciferol and boosting 25(OH)VD blood levels. Validation of this novel approach will lead to the design of new clinical trials using LC supplementation coupled with lower vitamin D doses as an adjuvant therapy to reduce 25(OH)VD deficiency/inadequacy and its associated complications and help reduce the related health hazards, particularly in the AA population.

## Figures and Tables

**Table 1:** Studies investigating the role of VD metabolism genes in obese humans.

Subject Subjects	Sample Size (*n*)	Purpose/Hypothesis	Outcome	Reference
Obese and lean adults	63 20 obese and 20 lean women 17 obese adults 6 lean women	VD-metabolizing enzymes were expressed differently in AT of lean and obese individuals and visceral adipose tissue (VAT) and subcutaneous adipose tissue (SAT), and their expression was influenced by weight loss.	VD-metabolizing enzyme expression differed within different ATs. *CYP27B1* ↓ in SAT of the obese. *CYP27A1* ↑ after weight loss.	[39]
Obese Italian men	121 54—non-obese 67—obese	To determine whether the trafficking of VD is altered in dysfunctional AT.	Dysfunctional AT shows a reduced catecholamine-induced release of D3 and 25(OH)D_3_ and altered activity of VD-metabolizing enzymes.	[40]
Obese Iranian patients	91 35—non-obese 33—morbidly obese 23—obese	To illustrate the determinants of VDR gene expression in visceral and subcutaneous adipose tissue among individuals without diabetes.	VDR ↓ in obese subjects and is negatively associated with 25(OH)D; positively associated with HOMA-IR.	[41]
Obese female patients and HFD mice	Human—4 women Mice—23 (M, 11; F, 12)	To explore the relationship between obesity and *CYP2R1* gene expression in human and mouse tissues.	*CYP2R1* expression is regulated by energy homeostasis in both humans and mice. *CYP2R1* ↓.	[10]
Hungarian adults	462 (M, 228; F, 234)	To investigate the relationship between BMI and genetic polymorphism of VD metabolizing genes.	Two SNPs in *CYP2R1* and VDR showed significant association with BMI.	[38]
Non-diabetic obese/overweight Brazilian adolescents	174 (MS, 48; non-MS, 126)	To investigate the associations of *CYP2R1* and *VDR* variants with MS and MS components in non-diabetic Brazilian adolescents.	SNPs are associated with increased risks of diabetes and hypertension in overweight/obese subjects. rs12794714 in *CYP2R1* is associated with MS and could be a possible new marker for predicting the risk of MS.	[42]
Obese Saudi women	100 (31 non-obese; 69 obese)	Testing the associations and the mechanisms involved in the silencing of the *CYP2R1* gene in normal and obese Saudi female patients.	Hypermethylation of specific sites in *CYP2R1* and *CYP27B1* regulates gene expression and is linked to obesity and VD metabolism.	[37]

AT—adipose tissue; HFD—high-fat diet; MS—metabolic syndrome; ↑, upregulation; ↓, downregulation.

**Table 2:** Studies investigating the role of VD metabolism genes in obese mice.

Mice/Treatment	Sample Size (*n*)	Purpose/Hypothesis	Outcome	Reference
HFD-induced obese mice and control mice	28 (14 per group)	To investigate the effects of HFD-induced obesity on VD metabolizing enzyme expression.	HFD-induced obesity influences VD-metabolizing enzyme expression, leading to abnormal regulation of serum 1,25(OH)2D. *Cyp2r1*, *Cyp27a1*, *Cyp2j3* ↓ in liver; *Cyp27b1* ↑, *Cyp24* ↓ in kidney.	[43]
HFD VD-deficient mice and control mice	25 (control, 7; 3 treatment groups, 6 each)	Glutathione stimulates VD regulatory and glucose-metabolism genes, lowers oxidative stress and inflammation, and increases 25(OH)VD levels.	HFD downregulates VD metabolism genes, VD+LC supplementation upregulates the gene expression and is a novel and better strategy to increase VD levels.	[44]
Female HFD and control mice	14 (5 per group)	To investigate the alternative mechanism that reduced the capacity to convert parent VD to 25(OH)D due to decreased expression of *Cyp2r1.*	*Cyp2r1* ↓ VD supplementation is less effective in obese subjects.	[11]
HFD and control mice	20 (10 per group)	Obesity disrupts VD homeostasis in key organs of VD metabolism.	Adipose tissue plays a vital role in the modulation of VD metabolism during obesity. Cyp2r1 induction is associated with low VD levels in adipose tissue.	[45]
HFD and control mice	19 (control, 10; HFD, 9)	Nutritional deprivation-responsive mechanisms regulate VD metabolism.	Both fasting and diabetes suppressed hepatic cytochrome P450 *Cyp2r1*.	[46]
HFD and control mice	4 per group	GSH deficiency induces epigenetic alterations of VD metabolizing genes, thereby reducing the circulating 25(OH)VD_3_ levels in obesity.	*Cyp2r1* ↓ in the mice liver. GSH is a potential adjuvant therapeutic target for normalizing 25(OH)VD_3_ status in vulnerable populations.	[47]
Obese and control mice	80 (20 per group)	To study the correlation of 25(OH)D3, physiological and pathological changes caused by obesity, and the motility of sperm.	*Cyp2r1* ↓ reduces the levels of 25(OH)VD, which interferes with regulating reproductive hormones.	[48]
HFD and control mice	56 (6 groups) Control and HFD with either LVd, CVd, or HVd	Low VD status in obesity decreases the bioavailability of VD to sequestration in adipose tissue.	Excess of body adiposity contributes to lower serum 25(OH)D levels.	[49]
High fat and high cholesterol diet mice and control mice	30 (10 per group)	Diet could impair VD metabolism.	HFD and HCD reduce serum 25(OH)D3 by suppressing hepatic *Cyp2r1* ↓.	[50]
HFD and control mice	20 (10 per group)	To investigate the impact of a short-term HFD on VD metabolism.	HFD-induced obesity decreases 25(OH)D and modulates gene expression in VD metabolism. *Cyp2r1*, *Cyp3a11* ↓ in the liver, *Cyp24a1*, and *Cyp27b1*↑ in the kidney of obese mice.	[51]

HCD—high-cholesterol diet; HFD—high-fat diet; ↑, upregulation; ↓, downregulation.
